# Attitudes of undergraduate health science students towards patients with intellectual disability, substance abuse, and acute mental illness: a cross-sectional study

**DOI:** 10.1186/1472-6920-10-71

**Published:** 2010-10-21

**Authors:** Malcolm J Boyle, Brett Williams, Ted Brown, Andrew Molloy, Lisa McKenna, Elizabeth Molloy, Belinda Lewis

**Affiliations:** 1 Monash University, Department of Community Emergency Health and Paramedic Practice, P.O. Box 527, Frankston 3199, Victoria, Australia; 2 Monash University, Department of Occupational Therapy, P.O. Box 527, Frankston 3199, Victoria, Australia; 3 Monash University, School of Nursing and Midwifery, P.O. Box 527, Frankston 3199, Victoria, Australia; 4 Monash University, Centre for Medical Health Science Education, Building 2, 270 Ferntree Gully Rd, Notting Hill 3168, Victoria, Australia; 5 Monash University, School of Primary Health Care, P.O. Box 527, Frankston 3199, Victoria, Australia

## Abstract

**Background:**

There is a long history of certain medical conditions being associated with stigma, stereotypes, and negative attitudes. Research has shown that such attitudes can have a detrimental effect on patients presenting with stigmatised medical conditions and can even flow on to impact their family. The objective of this study was to measure the attitudes of undergraduate students enrolled in six different health-related courses at Monash University toward patients with intellectual disability, substance abuse, and acute mental illness.

**Methods:**

A convenience sample of undergraduate students enrolled in six health-related courses in first, second and third years at Monash University were surveyed. The *Medical Condition Regard Scale *- a valid and reliable, self-report measure of attitudes - was administered to students along with a brief demographic form. Mean scores, t-tests, and ANOVA were used to analyse student attitudes. Ethics approval was granted.

**Results:**

548 students participated. Statistically significant differences were found between the courses (p = 0.05), year of the course (p = 0.09), and gender (p = 0.04) for the medical condition of intellectual disability. There was no statistically significant difference between the courses, year of the course, gender, and age group for substance abuse or acute mental illness conditions.

**Conclusion:**

The findings suggest that students in undergraduate health-related courses, as a group, have a strong regard for patients with intellectual disability and some regard for patients with acute mental illness, but not for patients presenting with substance abuse problems.

## Background

Historically, certain medical conditions (e.g. mental illness, physical disabilities, developmental disability) were isolated from mainstream society. Stigma, detracting stereotypes, and negative attitudes toward medical conditions are a major impediment in the provision of healthcare, with research showing that such attitudes can have a direct impact on patients' well-being and the type of health care they receive [1]. Students enrolled in health professional education programs are exposed to these patient groups, during their clinical placements, which they will be working with once they become qualified in their respective disciplines. Students may bring with them potential pre-conceived perceptions, notions, and values and then they become socialised to their chosen discipline [2,3]. Hence it is important to examine these attitudes, beliefs, and perceptions about the patient groups they will be working with.

Understanding the attitudes and beliefs towards different medical conditions amongst undergraduate students is a fundamental step in addressing the issue of negative attitudes so often reported in studies [4,5,1]. Once these students graduate they will be expected to treat a wide range of people presenting with various medical conditions without bias throughout their professional careers. It is therefore important to understand these attitudes and the effect their education has on attitudes in order to effectively promote and instil appropriate attitudes amongst our future healthcare professionals.

An attitude is a hypothetical construct that represents a person's degree of like or dislike for a place, person, thing, item or event. The term 'attitudes' is used to describe the pattern of feelings, beliefs, and reactions that an individual holds regarding particular people, objects, or ideas and are often formed based on an individual's past experience [6,7]. Attitudes are generally positive or negative views to things in the world around us. People can also be conflicted or ambivalent toward an object, situation, individual, or group, meaning that they simultaneously possess both positive and negative attitudes toward the item, issue, or person in question. Pre-existing negative attitudes have the potential to lead people to become closed-minded and biased in their interactions with the person for which the attitudes are held [2]. The manner in which an individual expresses an attitude is said to vary, with individual differences occurring in both the direction of the attitude or the strength and intensity with which a particular attitude is held [2]. Some attitudes may be expressed overtly while others may be more veiled, subtle, or covert.

Attitudes are judgments that develop on the ABC model (affect, behaviour, and cognition) [8]. The *affective *response is an emotional response that expresses an individual's degree of preference for an entity. The *affective *dimension incorporates the individual's particular feelings and emotions which underline the attitude, such as liking or disliking a particular person or group [8]. The *behavioural *intention is a verbal indication or typical behavioural tendency of an individual. The *behavioural *dimension reflects the observable reaction to an attitude, its function being to shape the action in response to the attitude. The *cognitive *response is a cognitive evaluation of the entity that constitutes an individual's beliefs about the object. The *cognitive *dimension relates to the thoughts, ideas, beliefs, or opinions associated with the attitude, allowing the person to mentally conceptualize, categorise, process, and remember the particular person or event for which the attitude is held [8,7].

Three other models have been developed that look at how people process messages and the impact of this on attitudes in an attempt to explain the affective (emotion) and cognitive processing, and interpretations of messages. These include the *Elaboration Likelihood Model *(ELM), the *Heuristic-Systematic Model *(HSM), and the *Extended Parallel Process Model *(EPPM). In the ELM cognitive processing is the central route and affective/emotion processing is often associated with the peripheral route [9]. The central route pertains to an elaborate cognitive processing of information while the peripheral route relies on cues or feelings. The ELM suggests that true attitude change only happens through the central processing route that incorporates both cognitive and affective components. This suggests that motivation through emotion alone will not result in an attitude change. In the HSM, information is either processed in a high-involvement and high-effort systematic way, or information is processed through shortcuts known as heuristics [10]. Emotions, feelings and gut-feeling reactions are often used as shortcuts. The EPPM, includes both thinking and feeling in conjunction with threat and fear appeals [11]. EPPM suggests that persuasive fear appeals work best when people have high involvement and high efficacy. In other words, fear appeals are most effective when an individual cares about the issue or situation, and that individual possesses and perceives that they possess the agency to deal with that issue or situation.

When negative attitudes are formed about a particular group of people, it is likely that they will be treated poorly, rejected and devalued within society. Furthermore, negative attitudes are often associated with people who have certain types of diagnoses or disabilities, leading the individual to experience limited lifestyle, educational and vocational opportunities, a decrease in overall quality of life and a decline in community participation [12,3]. Children and adults who are sick or present with certain health care problems commonly receive treatment and support from health professionals and it is assumed that health professionals are free from negative attitudes. However, positive and negative attitudes are formed routinely, and members of different health care disciplines are as susceptible to experiencing the same negative attitudes as individuals within society [8,2]. Negative attitudes held by health care professionals towards people with diagnoses can impact on the quality and range of services offered, and also hinders the development of the therapeutic relationship between the patient and health care provider [12,3]. The study of health professionals towards people with specific medical conditions has been documented in health literature; however there has been limited investigation into the attitudes held by health professional students' attitudes.

There have been three previous studies that have used the *Medical Condition Regard Scale *(MCRS) to determine attitudes of healthcare professionals towards patients with specific medical conditions [13]. The first study reported details of the MCRS's initial development. It was found that the MCRS was a valid and reliable measure of attitude (coefficient alpha = 0.87 and test-retest reliability = 0.84) [13]. The second study measured change in students' attitudes toward patients with a known history of substance abuse as a result of a one-week clinical placement experience in an addiction treatment facility [4]. The third study measured the change in attitude amongst a cohort of nursing students after a voice simulation experience toward patients experiencing auditory hallucinations [14]. Published studies into attitudes towards specific medical conditions for midwifery, health science, occupational therapy, physiotherapy, and emergency health (paramedics) undergraduate students has not previously been undertaken. The objective of this study was to measure the attitudes of undergraduate students enrolled in six different health-related courses at Monash University toward patients presenting with intellectual disability, substance abuse, and acute mental illness.

## Methods

### Design

A cross-sectional study using a paper based version of the *Medical Condition Regard Scale *(MCRS) questionnaire was administered.

### Participants

There were 1,296 undergraduate students eligible for inclusion in the study, see Table 1 for a breakdown of course, year numbers, and gender distribution. Students had to be undergraduates currently enrolled in one of six health-related courses at Monash University - emergency health (paramedic), nursing, midwifery, occupational therapy, physiotherapy, and health science.

**Table 1:** Student numbers by course by year

Course	Year 1	Year 2	Year 3	Total Number of students enrolled	Total Number of female students	Total Number of male students
Emergency Health (Paramedic)	12	77	160	249	165	84
Nursing	35	153	270	458	421	37
Midwifery	4	31	51	86	86	0
Occupational Therapy	18	33	118	169	143	26
Physiotherapy	13	42	181	236	161	75
Health Science	24	29	45	98	86	12

### Instrumentation

The *Medical Condition Regard Scale *(MCRS) was developed to provide a measure of attitudes that could be applied to any medical condition and allow for comparison between them [13]. This study utilised the MCRS (see Additional File 1) since it measures attitudes toward medical conditions and in particular whether students find such medical conditions to be enjoyable, treatable and worthy of medical resources. The MCRS is designed such that it can be used with any medical condition. This provides a useful degree of flexibility allowing the health-related courses involved in this study to apply additional medical conditions most relevant to their professional field, hence not all disciplines have results for all three conditions in Table 2. The MCRS is considered valid and reliable and its authors found the scale to have a Cronbach coefficient alpha of 0.87 and a test re-test reliability of 0.84 [13]. It is, however, a self-report questionnaire and this needs to be kept in mind in the interpretation of the results as it measures what students report rather than what are necessarily their actual attitudes.

**Table 2:** Mean MCRS result for the conditions by health-related course

Medical Conditions	Emergency Health (Paramedic) mean(SD)	Nursing mean (SD)	Midwifery mean (SD)	Occupational Therapy mean (SD)	Physiotherapy mean (SD)	Health Science mean (SD)
Intellectual Disability	55.28(8.14)	53.88(9.41)	53.51(10.17)	N/C	51.10(8.77)	55.31(8.98)
Substance Abuse	47.47(9.22)	48.37(10.30)	44.46(12.48)	47.10(9.22)	N/C	49.84(7.53)
Acute Mental Illness	54.92(9.96)	53.94(9.53)	51.93(9.99)	N/C	N/C	56.65(8.24)
**Total students**	**119**	**107**	**52**	**92**	**109**	**69**

Legend: N/C = Not Collected

The eleven items on the MCRS were rated on a 6-point Likert scale (1 = strongly disagree, 6 = strongly agree). To reduce the confounding effect of acquiescent responding, five of the eleven items are worded negatively, which are later reversed-scored for analysis. As such, the closer a mean score is to six, the more indicative it is of positive regard/attitude toward that medical condition being measured.

The three patient conditions included as part of the MCRS were intellectual disability, substance abuse, and acute mental illness. In the context of this study, acute mental illness was defined as an episode of the patient's current mental health condition requiring the attendance of an ambulance and hospitalisation for ongoing management.

### Procedure

Students received an explanatory statement outlining the purpose of the study beforehand and were informed that participation was voluntary and anonymous. Each participant completed a self-report questionnaire including a brief set of demographic questions and the MCRS. The questionnaire was completed at the end of a lecture for each respective group of health-related students and took approximately 10 minutes to complete. A non-teaching member of staff facilitated the process and collected the questionnaires. Consent was implied by completion of the questionnaire.

### Ethics

Ethics approval for the study was granted by the Monash University Standing Committee on Ethics in Research Involving Humans (SCERH).

### Data analysis

Descriptive and inferential data analysis was undertaken using SPSS (Statistical Package for the Social Sciences Version 17.0, SPSS Inc., Chicago, Illinois, U.S.A.). Descriptive statistics, means and standard deviations, were used to summarise the demographic and some MCRS data. Inferential statistics, t-test and ANOVA, including post hoc (Sidak or Tamhane) tests where required, were used to compare the differences between courses, age groups, gender, and year of the course. All tests were two tailed unless otherwise stated with the results considered statistically significance if the p value is < 0.05.

## Results

### Overview

A total of 548 undergraduate students participated in this study, with student representation from the six health-related courses. The number of students from each course, the medical conditions and the students regard for those medical conditions are presented in Table 2. Because convenience sampling was used, data was not available on the number of students who declined to participate and consequently no response rate could be calculated.

The majority of students were female (n = 439, 81%) which is in line with the total number available for inclusion (82%), see Table 1. The majority of students were <25 years of age (n = 436, 80%). Each year level was adequately represented for statistical analysis, 1^st ^year (n = 135, 24.6%), 2^nd ^year (n = 234, 42.7%), 3^rd ^year (n = 179, 32.7%). There was also a strong internal consistency for the MCRS as measured by Cronbach's Coefficient Alpha of 0.93.

### Intellectual Disability Group

Overall intellectual disability as a medical condition was held in high regard by the students (mean = 53.76, SD = 9.07). There was a statistically significant difference in the attitude reported towards intellectual disability as a medical condition between the six courses (p = 0.005), with students studying physiotherapy having reported the lowest mean regard (mean = 51.10, SD = 8.77), see Table 2. Statistically significant differences were also reported between year level (p < 0.009) and gender (p < 0.004). The notable differences within these two variables were that third year students reported the lowest regard (mean = 51.99, SD = 9.34) and females reported the highest regard (mean = 54.51, SD = 8.91) compared to male students.

On the MCRS, students rated their level of agreement on a 6-point scale for each item and mean item scores below 3.5 are therefore indicative of negative regard or attitude. For intellectual disability, there were no negative means for each course on any individual scale item. There were, however, three items which received notably high means overall. From highest to lowest they were 'Treating patients like this is a waste of medical dollars' (mean = 5.87), 'Patients like this irritate me' (mean = 5.60) and 'There is little I can do to help patients like this' (mean = 5.48).

### Substance Abuse Group

Substance abuse as a medical condition was held in the lowest regard by midwifery students (mean = 44.46, SD = 12.48). The means for each health-related course are presented in Table 2. Females (mean = 47.82, SD = 9.89) had only a marginally more positive attitude to patients with substance abuse problems than males (mean = 47.25, SD = 8.04) with third year students having the least positive attitude (mean = 46.97, SD = 8.73).

Results from the MCRS's individual items provide more detail as to how students reported their regard for patients with substance abuse problems. Slightly negative mean scores were obtained for three items on the MCRS, 'I feel especially compassionate toward patients like this' (mean = 3.36), 'I wouldn't mind getting up on call nights to care for patients like this' (mean = 3.35) and 'I enjoy giving extra time to patients like this' (mean = 3.31).

Despite some negative regard, three items received means indicative of a positive regard towards substance abusing patients. They were: 'There is little I can do to help patients like this' (mean = 5.46), 'Patients like this irritate me' (mean = 5.00) and 'Treating patients like this is a waste of medical dollars' (mean = 5.45).

### Acute Mental Illness Group

There was some regard identified to patients with acute mental illness as a medical condition by the students. There was no statistically significant difference with respect to mean regard for acute mental illness was by course, year, gender or age group. Students enrolled in emergency health (paramedics) reported the highest mean regard for acute mental illness (mean = 54.92, SD = 9.96), see Table 2.

The MCRS item that received the highest mean with regard to acute mental illness was 'Treating patients like this is a waste of medical dollars' (mean = 5.81). Two other items that received particularly high mean scores were: 'There is little I can do to help patients like this' (mean = 5.41) and 'I prefer not to work with patients like this' (mean = 5.46). An item which would have received a mean score indicative of high regard if students enrolled in health science were not included was: 'Patients like this irritate me' (mean = 4.87). Students enrolled in health science reported an extremely low regard for this item (mean = 2.36).

## Discussion

In this study the findings demonstrate that students from the six different health-related courses have a high regard for the medical conditions of intellectual disability and moderate regard for patients with acute mental illness. Substance abuse, however, received a larger number of negative responses.

Most studies into stigma and attitude use research methodologies tailored specifically for the medical conditions being investigated. As such, the results from most other studies are not directly comparable or generalisable. Furthermore, given that attitudes toward medical conditions are culturally and socially sensitive, caution is required when comparing results as they may not be applicable to the present context in Australia. A good example of how attitudes change is shown in Tierney's review on the knowledge, attitudes, and education of nurses with regard to HIV/AIDS in the United Kingdom (UK). In the mid-1980s Tierney reported that nurses were fearful of AIDS, but that by the time her literature review was conducted in 1995, there had been a 'significant improvement in nurses' knowledge and attitudes' toward HIV/AIDS. The change in attitude towards patients with HIV/AIDS was brought about by factual education programs [15]. In other words, there was a significant shift in attitudes and perceptions of members of the nursing profession towards this medical condition following an education program targeting all health care providers.

An analysis of the MCRS's items show in more detail where the difference lies. Across all the medical conditions, students answered in such a way that was indicative of a desire to act professionally. Items such as 'Patients like this irritate me' and 'Treating patients like this is a waste of medical dollars' were answered favourably across all three medical conditions, showing an intention to be fair and responsible in their conduct. The item 'I feel especially compassionate toward patients like this' differed between the medical conditions and was particularly low for substance abuse (mean = 3.36) compared to intellectual disability (mean = 4.25) or acute mental illness (mean = 4.29). Because items were rated on a 6-point Likert scale scores below 3.5 are indicative of negative attitudes. As such, a mean of 3.36 regarding compassion toward patients who have a history of substance abuse is negative. The other three items that come very close to or actually receive negative means with regard to substance abuse were 'I wouldn't mind getting up on call nights to care for patients like this' (mean = 3.35), 'I can usually find something that helps patients like this feel better' (mean = 3.60) and 'I enjoy giving extra time to patients like this' (mean = 3.31). These items suggest students have a reluctance to go out of their way for such patients and perhaps to a certain extent consider their skills not relevant to the problems faced by patients who have substance abuse problems.

Statistically significant differences were also found between the six health-related courses, specifically related to the intellectual disability condition. These differences may be the result of how students from each of the professions view their role in health care and the vantage their profession provides from which to view patients. As well, the differences between the health discipline groups may be attributed to the affective dimension of attitudes that incorporates feelings and emotions which underline the attitude, such as liking or disliking a particular person or group [8]. For instance, those students enrolled in midwifery reported the lowest regard for the substance abuse medical condition. These students possibly considered the effect substance abuse has on the foetus/baby as well as the substance abusing mother and, as such, were less compassionate toward such patients because of the effect certain substances such as alcohol have on the foetuses/baby's health.

Another example is students enrolled in the health science course. Their regard for patients suffering acute mental illness was consistent with the other professions, however, for the one item 'Patients like this irritate me' they were entirely consistent with the other student groups by reporting a particularly high regard. This result is, to a certain extent, inexplicable unless it is attributed to something from the health science course. Further medical conditions are likely to raise further differences between the student groups, although based on such data alone such inferences cannot be supported with absolute certainty. In any case, the data provides sufficient evidence to show that there is a difference in views between the health professional student groups and, more importantly, that the views these students have impact on their attitude toward different medical conditions.

A study by Abed and Neira-Munoz into general practitioners' attitudes towards patients with substance abuse problems determined that their negative attitudes were due to the fact that they considered such a medical condition to be a heavy burden and largely self-inflicted [16]. The students in this study did not give answers indicating they thought such patients were a heavy burden; however, they did answer as though their problems were self-inflicted and thus not as worthy of compassion or empathy. Considering the results of two other medical conditions included in this study provides further evidence for this point. Students enrolled in the occupational therapy course had a high regard for patients with depression (mean = 53.37, SD = 7.32). This is in stark contrast to patients with substance abuse problems, despite one of the symptoms of depression being a dependence on alcohol or drugs [17]. Furthermore, those students enrolled in the physiotherapy course reported their attitudes toward the medical condition of obesity, the only other condition included in the study that could be perceived to be self-inflicted or a result of personal choices made by individuals, as low as substance abuse (mean = 43.65, SD = 7.42). These findings suggest a marked difference in the regard toward a medical condition based on whether the student perceives the condition to be caused by patients' behaviour or not.

There is a wide variety of substances which can be abused, and they generally all have an associated stigma and negative perception. In addressing this problem, health care professionals play an important role in detection and intervention efforts [18]. However, a study by Abouyanni et al. of general practitioners in Sydney, Australia, found many lacked the confidence and experience necessary to deal with such patients and were worried that these patients would be 'difficult, aggressive and demanding' [19]. Another study of general practitioners almost two decades earlier in the UK found attitudes toward individuals with a history of substance abuse were negative, stemming from views that such patients were a heavy burden and their problems self-inflicted [16].

A number of investigations have found that such attitudes are unhelpful. A UK study looking into what can be done to help injecting drug users benefit from services highlighted the need to address negative preconceptions among professionals in that such attitudes have a negative impact on the treatment process [20]. In a study of those diagnosed with both having a history of substance abuse and having a mental illness such negative attitudes were found to further complicate the lives of those who have been stigmatised, even well after treatment had been successful [1]. Substance abuse is potentially an end-point, for example, a person with depression may take an overdose of their medication as they are unable to cope. Therefore, students need to understand this point and be prepared to identify the underlying cause, if any, behind the substance abuse and not just see it as "attention seeking" behaviour.

Persons with mental illness face similar issues with the impact that negative attitudes and stigma have on their well-being. A survey of individuals with mental illness in Australia found 74% of respondents had experienced stigma, with 13% having experienced stigma directly from health care service providers [21]. The same survey also found respondents would 'feel better about themselves' and 'manage their illness better' if stigma were reduced [21]. Indeed the consequences of stigma are numerous with a sense of shame and difference to others being two of the more apparent consequences [22]. Research has also found the effects of stigma are not confined to the individual, but extend to patients' family members as well [23].

What makes stigma and negative stereotyping difficult to combat is that some of the concerns people have about the persons with mental illness often have a partial basis in reality [5]. As Gray points out, such patients are not necessarily easy to 'like or engage with' but with better knowledge of their problems generally comes a less stigmatising approach [24]. An example of this is found in Fisher's study of a group of nursing students' first clinical placement at a mental health clinic [25]. The experiences of these students were predominately negative. It was suggested that the experience challenged the nursing students' image of what being a nurse involves because patients in certain cases were aggressive toward them or harassed them [25].

People with an intellectual disability may also exhibit behaviours that contribute to stigma, including challenging behaviour and sexualised behaviour. People with an intellectual disability lack the mental capacity to understand that certain behaviours are unacceptable, inappropriate, or unlawful in our society and thus this condition has a history of being linked to crime [26]. It is important that health care professionals understand how such behaviour comes about in people with an intellectual disability. There have been a number of studies which have shown that staff reactions to challenging behaviour in these people can have important implications on the intervention strategies employed to mitigate such behaviour [27]. There are also concerns about the poor healthcare that people with an intellectual disability receive. Such concern prompted one study of general practitioners which uncovered that while general practitioners were willing to treat people with an intellectual disability they were not willing to spend more time or complete additional training necessary to effectively treat this client group [28]. Another study found the attitude toward and comfort with people with an intellectual disability is potentially improved by spending time with this group [29].

The findings from this study can be extrapolated with some confidence to the broader student body enrolled in any of the courses that participated in this study, even though convenience sampling was used. The study sample included a majority of young, female adults, which is consistent with the actual gender make-up of most health-related courses (see Table 1). These results report students' attitudes toward the various medical conditions, but the findings do not confirm without doubt why students reported such a level of regard. The MCRS's items give direction to why students reported their level of regard, but further exploration is required to understand in detail what it is that makes, for instance, students view depression in a much more positive way compared to substance abuse. Further investigation into why such attitudes are held may also provide further insight into why there are statistical significant differences in attitudes between students in the six health-related courses.

This study is potentially limited by the use of convenience sampling of students from one specific educational institution. While this method facilitates recruitment, it is less likely to recruit a representative sample of students. Consequently, those students who did volunteer to participate may themselves bias the results. We are not able to calculate the distribution of non-respondents because none of the courses collect student attendance at lectures. Therefore we are unable to identify who is missing from the lecture at the time the questionnaire was administered and which students did not attempt the questionnaire. As well, ethically the investigators did not collect personal data from respondents since the questionnaires were completed anonymously. Caution is required when interpreting the results as it needs to be kept in mind that the MCRS is a self-report questionnaire and there is the possibility that respondents answered items in a 'socially desirably' manner. Another issue to consider is that the majority of the respondents were female; however, the gender distribution of the respondent group was representative of the students who are enrolled in health professional programs at Monash University. Therefore results of the students' reported views and perceptions may differ from their actual attitudes either in private or when confronted with a patient presenting with one of these medical conditions.

## Conclusion

This study has demonstrated that students have a reasonably strong regard toward patients presenting with intellectual disability and some regard for patients with acute mental illness. The results also suggest that where students perceive a medical condition to be self-inflicted or due to the patients personal choices, such as substance abuse, their regard declines significantly. Students' answers show an intention to remain professional despite their opinions for certain types of patients. Regard was lacking in students' compassion and willingness to go beyond professional expectations. The role students see themselves playing in the healthcare system also seems to have an impact on the perception of these medical conditions, showing that attitudes toward these medical conditions are not based solely on the condition being suffered. There is also a need to better educate the students, especially for patients presenting with substance abuse, as this may be a result of another condition like depression. The findings from this study also provide important information in curriculum renewal and the integration of teaching and research in each of health care professions.

## Competing interests

The authors declare that they have no competing interests.

## Authors' contributions

MJB, BW, TB conceived the idea of the study. MJB and AM undertook the analysis of the data. All authors interpreted the data and undertook a literature review for their discipline; each author contributed to writing the manuscript and has approved the submitted version.

## Pre-publication history

The pre-publication history for this paper can be accessed here:

http://www.biomedcentral.com/1472-6920/10/71/prepub

## Supplementary Material

Additional File 1 **Medical Condition Regard Scale **An example of the Medical Condition Regard Scale Click here for file
