# Supplementary material for: Attitudes of undergraduate health science students towards patients with intellectual disability, substance abuse, and acute mental illness: a cross-sectional study
Source: BMC Med Educ. 2010 Oct 21;10:71. doi: 10.1186/1472-6920-10-71 (PMC2972293; doi:10.1186/1472-6920-10-71)
Supplement: Additional File 1 — Medical Condition Regard Scale An example of the Medical Condition Regard Scale [file 1472-6920-10-71-S1.DOC]

Use the scale below to rate your degree of agreement or disagreement with each of the following items regarding patients from **[Medical Condition]:**

| **A** | **B** | **C** | **D** | **E** | **F** |
| --- | --- | --- | --- | --- | --- |
| Strongly Disagree |  |  |  |  | Strongly Agree |

|  |  | **A** | **B** | **C** | **D** | **E** | **F** |
| --- | --- | --- | --- | --- | --- | --- | --- |
| **1.** | Working with patients like this is satisfying. |  |  |  |  |  |  |
| **2.** | Insurance plans should cover patients like this to the same degree that they cover patients with other conditions. |  |  |  |  |  |  |
| **3.** | There is little I can do to help patients like this. |  |  |  |  |  |  |
| **4.** | I feel especially compassionate toward patients like this. |  |  |  |  |  |  |
| **5.** | Patients like this irritate me |  |  |  |  |  |  |
| **6.** | I wouldn’t mind getting up on call nights to care for patients like this |  |  |  |  |  |  |
| **7.** | Treating patients like this is a waste of medical dollars. |  |  |  |  |  |  |
| **8.** | Patients like this are particularly difficult for me to work with. |  |  |  |  |  |  |
| **9.** | I can usually find something that helps patients like this feel better. |  |  |  |  |  |  |
| **10.** | I enjoy giving extra time to patients like this. |  |  |  |  |  |  |
| **11.** | I prefer not to work with patients like this. |  |  |  |  |  |  |
